# Evaluation of rheological, textural, and sensory characteristics of optimized vegan rice puddings prepared by various plant‐based milks

**DOI:** 10.1002/fsn3.3872

**Published:** 2023-11-30

**Authors:** Atefeh Karimidastjerd, Zehra Gulsunoglu‐Konuskan, Emine Olum, Omer Said Toker

**Affiliations:** ^1^ Food Engineer/Researcher (a PhD degree in Food Engineering from Istanbul Technical University) Istanbul Turkey; ^2^ Faculty of Health Sciences, Nutrition and Dietetics Department Istanbul Aydin University Istanbul Turkey; ^3^ Faculty of Fine Arts, Design and Architecture, Department of Gastronomy and Culinary Arts Istanbul Medipol University Istanbul Turkey; ^4^ Faculty of Chemical and Metallurgical Engineering, Food Engineering Department Yildiz Technical University Istanbul Turkey

**Keywords:** plant‐based milks, rice pudding, RSM, vegan

## Abstract

Nowadays, plant‐based milks are being considered as an alternative to dairy milk due to their advantages, such as sustainability, reduced allergenicity, health benefits, and lactose‐free nature. Plant‐based milks are widely used in the preparation of desserts, cheese‐like products, and beverages, among other applications. The aim of the present study was to formulate vegan rice puddings using various commercially available plant‐based milks as a sustainable alternative to dairy milk. For this aim, central composition design was applied to optimize the key processing parameters of the Thermomix®, including temperature (80–90°C), time (6–14 min), and the amount of rice flour (6–10%, w/v), using response surface methodology (RSM). According to the RSM results, the optimum conditions were found to be 90°C for 12.5 min with 6.5% rice flour, as they exhibited minimal phase separation and similar rheological and textural properties to dairy rice pudding. Soya milk pudding had the highest hardness value among the other plant‐based milk puddings, and whole fat milk, soya, oat, coconut, and cow's milks showed the best gel unity, according to the cohesiveness results. Phase separation, an important parameter for storage stability, was not observed during 7‐day storage at 4°C in all groups, except for pistachio milk rice pudding. Rheological results demonstrated that all vegan pudding samples exhibited a gel‐like structure with storage modulus (*G′*) exceeding loss modulus (*G″*) values. According to the descriptive sensory evaluation, coconut, oat, and soya milk rice puddings received the highest scores in overall acceptability. Our findings suggest that industrial plant‐based rice puddings have great potential as a novel product that meets the dietary needs of the vegan community by offering acceptable flavor and texture.

## Graphical abstract

The aim of our study was to investigate structural, chemical, and sensory properties of rice puddings which were prepared with different plant‐based milks instead of whole fat and low‐fat milk.

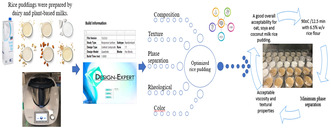

## 1 INTRODUCTION

Milk has traditionally served as a base material for the production of various dairy and food products, including cream, ice cream, yogurt, cheese, butter, and milk‐based desserts. However, a range of health issues, primarily affecting adults, such as dietary restrictions, allergies, and lactose intolerance, have resulted in the need to limit milk consumption in individuals (Plamada et al., 2023). Additionally, ethical concerns related to animal welfare and sustainability in food production systems have underscored the importance of reducing milk consumption and seeking alternatives to dairy milk (Astolfi et al., 2020; Karimidastjerd & Kilic‐Akyilmaz, 2021; McClements, 2020). Plant‐based milks are produced from a variety of pseudo‐cereals, seeds, legumes, nuts, and grains manufactured by mixing with water (Sethi et al., 2016). Common ingredients for plant‐based milks include soya, almonds, rice, oats, hazelnuts, and coconut. Less common sources, though occasionally used, include hemp, pistachio, macadamia nuts, flax, and spelt (Karimidastjerd & Gulsunoglu‐Konuskan, 2021; Karimidastjerd & Kilic‐Akyilmaz, 2021). The primary drawback of plant‐based milks, when compared to dairy milk, is the absence of many milk minerals and vitamins, necessitating the fortification of commercial plant‐based milks. Currently, commercial plant‐based milks are readily available in most markets in various countries (Karimidastjerd & Gulsunoglu‐Konuskan, 2021).

Milk serves as a primary ingredient in many desserts, and among milk‐based desserts, puddings have gained widespread popularity worldwide. Puddings have a typical semi‐solid texture resulting from the interaction of milk proteins and starch or other hydrocolloids (Alamprese & Mariotti, 2011; Alamri et al., 2014; Chandan & Kilara, 2008). They are typically prepared by cooking a mixture of flour, sugar, milk, and vanilla, and ingredients such as eggs, starch, rice powder, gelatine, hydrocolloids, flavorings, and colorings can vary by region and consumer desire (Nugroho et al., 2018; Tarrega & Costell, 2006). Fereni, a traditional Iranian rice pudding, is made with dairy milk, rice flour, rosewater, and sugar in its original recipe. It is known for being gluten‐free and nutritious compared to other starch‐ and sugar‐based desserts (Gharibzahedi, 2018). Similarly, the traditional Turkish dairy dessert Muhallebi is made with dairy milk, starch, and sugar, without the use of hydrocolloids (Okur, 2023). Various rice varieties, categorized by color (red, white, black, brown, and purple), have been studied for their potential as antioxidants and functional ingredients (Goufo & Trindade, 2014).

While Fereni and Muhallebi are traditionally prepared with dairy milk, vegan alternatives are not yet available in commercial markets. The development of new products, including legume‐based options, is the focus of several studies, with a strong emphasis on evaluating consumer acceptance (Ng'ong'ola‐Manani et al., 2014). However, a key challenge in the widespread use of plant‐based milk puddings is the lack of information on the best processing conditions to achieve optimal texture and rheology. Limited data exist on the substitution of milk with plant‐based sources (Alamprese & Mariotti, 2011; Lim & Narsimhan, 2006; Nunes et al., 2003). For example, Alamprese and Mariotti (2011) examined the substitution of dairy milk with rice and soya drinks in the presence of carrageenan, evaluating the texture and viscoelastic behavior of puddings. Nunes et al. (2003) used plant‐based protein isolates, such as pea isolate, lupin isolate, and soya isolates, in combination with xanthan, k‐carrageenan, gellan gum, and maize starch, to study the texture and rheological properties of pudding. In another study conducted by Lim and Narsimhan (2006), soya protein concentrate was used as a dairy substitute for pudding preparation, and the rheological behavior of the pudding was examined.

The discovery of the multifunctional food processor Thermomix® in 1961 triggered a huge amount of innovative culinary recipes and provided a way to prepare more standardized foods with less time consumption. Thermomix® offers a wide range of functions, including cutting, chopping, blending, mixing, weighing, grinding, kneading, steaming, beating, stirring, emulsifying, and cooking at the desired temperature, mixing speeds, and time (Amend & Cavagnaro, 2021). Thermomix® is essential in modernist restaurant kitchens (Lavelle et al., 2021), and numerous scientific research studies have explored its application in food preparation (Bähler et al., 2016; Freire et al., 2016; Kolniak‐Ostek et al., 2013; Müller et al., 2017; Velázquez et al., 2021; Zargaraan et al., 2015). System optimization for a specific food product is important for the widespread use of Thermomix®, which has accepted modern and timely efficient food processing techniques. Response surface methodology (RSM) is a powerful tool for testing multiple variables and identifying interactions between different factors (Ghasemlou et al., 2012).

The aim of this study is to develop vegan rice puddings using different plant‐based milks (almond, soya, coconut, oat, and pistachio) with Thermomix® and compare them to cow's milk‐based puddings. The study evaluates processing conditions, including temperature (°C), time (min), and rice flour content (%, w/v), using RSM. This research also aims to fill the gap in vegan recipes by determining the optimal process conditions for plant‐based puddings from various sources, achieving the best rheological and textural properties while maintaining acceptable sensory characteristics.

## 2 MATERIALS AND METHODS

### 2.1 Materials

All ingredients used for pudding preparation were commercial products procured from a local supermarket in Istanbul, Türkiye. Eight different types of commercially available UHT plant‐based milks (low‐fat (1.5%) and whole‐fat (3%) cow's milk, almond, oat, Antep pistachio, coconut, soya, and hazelnut plant‐based milks) were used, and all puddings contained rice flour and sugar. The chemicals used in this study, including sodium hydroxide (NaOH), boric acid (H_3_BO_3_), sulfuric acid (H_2_SO_4_), copper (II) sulfate (CuSO_4_.5H_2_O), potassium sulfate (K_2_SO_4_), methyl red, brom cresol green, ethyl alcohol, and petroleum ether, were of analytical grade and were obtained from Merck Chemicals (Darmstadt, Germany).

### 2.2 Experimental design and optimization

Pudding formulations and different cooking conditions in a food processor significantly influence the textural and sensory properties of the final product. Therefore, it is important to optimize the process conditions and pudding formula to achieve an acceptable dessert. To determine the optimal process conditions, whole‐fat cow's milk and oat milk were selected as representatives for all types of milk to produce rice pudding. Oat milk was selected as the plant‐based milk for the optimization process due to its cost‐effectiveness compared to other plant‐based milk alternatives. RSM was employed to optimize the process conditions using Design Expert version 10 (Stat‐Ease, MN, USA). Three independent variables were chosen, two being process variables and one a composition variable. The levels of these variables were determined based on our preliminary results (data now shown). Three independent factors, including temperature (°C), time (min), and rice flour (w/v%), were set according to the Central Composite Design (CCD) as outlined by Karimidastjerd (2020). No blocks were used as all samples were prepared on the same day, resulting in 17 experimental runs for both whole milk and oat milk rice puddings (Table 1, 2). CCDs are a factorial design with center points, augmented by a group of axial points, also known as star points, which enable the estimation of curvature, as demonstrated by Ghasemlou et al. (2012). To determine the lower and upper limits of temperature, time, and rice flour, we conducted preliminary tests, and after that, seventeen experiments were designed, including three replications at the center point, to evaluate the *p* value, *R* ^2^, model equations, and the relationship between the independent factors and the responses (dependent factors). Responses included phase separation on the 1st and 7th days after production and the rheological (*G′*, *G″*, and G*) properties. Optimum conditions were determined based on the rheological properties of commercially available milk‐based rice pudding, and these conditions were then applied to produce rice puddings using five different plant‐based milks and two kinds of cow's milk (whole and low‐fat).

### 2.3 Pudding preparation

Rice puddings were prepared by combining milk and solid ingredients, which included 6% (w/v) sugar for all samples and rice flour at varying concentrations of 6%, 8%, and 10% (w/v), in a Thermomix® (TM5, Vorwerk, Germany) set at 1200 rpm (setting 3). The mixture was prepared in a closed container to prevent evaporation. The optimization conditions were chosen with independent factors (time, temperature, and the amount of rice flour) within a specified range, aiming to achieve rheological properties and minimal phase separation in the final product based on the characteristics of a commercial rice pudding (Turkish Muhallebi). The commercial rice pudding exhibited 0% phase separation on both the 1st and 7th days and had recorded values of 62.17 Pa for *G′*, 24.17 Pa for *G″*, and 54.76 Pa for G*.

According to the optimization results, the optimal values for cooking time, temperature, and rice flour amount were determined to be 12.5 min, 90°C, and 6.5%, respectively, for both plant‐based and cow's milk variations. After the cooking process, the samples were allowed to cool to room temperature and then stored at 4°C overnight. The day after production, a comprehensive analysis was performed, including proximate composition, color, phase separation, rheological and textural properties, and sensory evaluation of the rice puddings. Pudding production was conducted in duplicate, and each sample was subjected to three separate analyses.

### 2.4 Proximate composition

The rice puddings were subjected to analysis for total protein, fat, ash, dry matter, and carbohydrate content. The protein content was determined using the macro Kjeldahl method (AOAC, 2002), where the nitrogen content was calculated using conversion factors: 5.18 for nut and seed milk, 5.83 for oat milk, 5.95 for rice milk, 5.71 for soya milk, and 6.38 for bovine milk, following the guidelines of WHO/FAO (Jeske et al., 2017). The ash content was calculated through gravimetric methods based on the AOAC (1990) procedure. To assess the dry matter content, the rice pudding samples were subjected to drying in a vacuum dryer (Nüve EV018, Ankara, Türkiye) at 70°C, and moisture was determined gravimetrically (AOAC, 1995). The total fat content was evaluated using the Soxhlet method, as outlined in AOAC (2000). Total carbohydrate content was calculated by subtracting the values of protein, fat, and ash from the total dry matter (Hendek Ertop et al., 2019). The pH value of the samples was measured using a pH meter (Mettler Toledo, Istanbul, Türkiye).

### 2.5 Rheological properties

Rheological measurements were conducted using a rheometer (Rheostress 1, Thermo Electron GmbH, Karlsruhe, Germany). The measurements were performed at 10 ± 1°C using a parallel plate probe with a 35 mm diameter (C35/Ti, Thermo Electron GmbH) and a 1 mm gap size. To determine the linear viscoelastic region, a stress sweep analysis was carried out over a range of 0.1–10 Pa. Additionally, a frequency sweep test was conducted within a frequency range of 0.1–100 Hz at 0.5 Pa (within the linear viscoelastic region) and at a temperature of 10 ± 1°C. The storage modulus (*G′*), loss modulus (*G″*), and complex modulus (G*) were calculated using the rheometer software (RheoWin V.3.3, Haake, Karlsruhe, Germany) as described by Toker et al. (2013).

### 2.6 Texture analysis

Texture analysis was performed using a texture analyzer (TA Plus Texture Analyzer; Lloyd Instruments, Fareham, United Kingdom) following the method by Ngamlerst et al. (2022) with some modifications. Pudding samples (100 g) were weighed and stored overnight at 4°C in a 150 mL cup to allow the samples to reach equilibrium. A cylindrical probe with a 25‐mm diameter was inserted into the cylindrical cup. The probe's velocity and trigger settings were 0.5 mm/s and 1.5 gf, respectively. The deformation of the pudding samples was maintained at 50% of the total compression, reaching a depth of 10 mm. Various textural parameters were determined, including hardness, which represents the maximum force during the first compression (*N*); cohesiveness, calculated as the ratio of the area under the first and second compressions; and gumminess, derived from the hardness times cohesiveness (g). Each sample was analyzed thrice.

### 2.7 Phase separation

To evaluate phase separation, 10 mL of pudding samples were poured into 15 mL graduated tubes and then stored at 4°C. Measurements were carried out on both the 1st and 7th days, and the quantity of separated water at the top of the tubes was recorded as the height (mL). Phase separation was expressed as a percentage (%) of the total height (Karimidastjerd & Kilic‐Akyilmaz, 2021).

### 2.8 Instrumental color parameters

To measure the color parameters of the pudding samples, a colorimeter (CR‐400, Konica Minolta Inc., Tokyo, Japan) was used after calibrating the instrument with a standard white plate. The color parameters for the standard white plate were 29.94 for L* (lightness/darkness), 7.29 for a* (red/green), and 12.09 for b* (yellow/blue). The measurement process involved placing a clear plastic cup containing the pudding samples on the top of the colorimeter. The browning index (BI) was calculated using the following formula, as specified by Maskan (2001).

$$\mathrm{BI}=\frac{100\left(x-0.31\right)}{0.17}$$

$$x=\frac{\left(a^{*}+1.75L^{*}\right)}{5.645L^{*}+a^{*}-3.012b^{*}}$$

### 2.9 Sensory analysis

All pudding samples were analyzed for particle size, particle structure, viscosity, color, smell, taste, flavor, and overall acceptability after 1 day of storage at 4°C. The descriptive sensory evaluation method, utilizing a 6‐point scale, was employed to assess the particle size (0 = like flour–5 = like sugar crystals), particle structure (0 = like milk‐5 = like soured milk), consistency (0 = like water‐5 = like honey), and color (0 = creamy‐5 = white) properties of the samples, involving the participation of 10 trained panelists (Lawless & Heymann, 2010). A 6‐point hedonic scale was provided to the panelists, ranging from 0 (none) to 5 (extreme) for the other parameters (taste, aroma, smell, and general acceptability). The sensory evaluation was repeated twice with the same panelists.

### 2.10 Statistical analysis

The Design Expert 11 program (Minneapolis, MN, USA) was used for RSM and optimization analysis. The average data obtained from three replications of measurements were input into Design Expert to identify the statistically significant independent factors (time, temperature, and rice flour amount) and their effects on the responses (rheology and phase separation parameters). Under optimized conditions, the SPSS® (IBM® Statistics 26, Armonk, NY, USA) program was used to compare the differences among the seven prepared rice pudding samples. For sensory analysis, data from three replications were subjected to a one‐way analysis of variance (ANOVA) using SPSS®. Tukey's multiple comparison test was employed to differentiate means at a 5% level of significance.

## 3 RESULTS AND DISCUSSION

### 3.1 Optimization of process parameters by response surface methodology

CCD was used to determine the optimum levels of temperature (X_1_), time (X_2_), and rice flour (w/w%) (X_3_) concerning rheological parameters and phase separation after the 1st and 7th days of storage. As part of this, oat milk (Table 1) and whole‐fat cow's milk (Table 2) were selected as the model systems. An analysis of variance (ANOVA) was conducted to assess the suitability of the proposed models and identify the significant levels.

**TABLE 1:** Experimental design and data under various conditions of oat milk rice pudding.

Run	Independent variables			Responses				
	*X*_1_	*X*_2_	*X*_3_	*Y*_1_	*Y*_2_	*Y*_3_	*Y*_4_	*Y*_5_
1	85	17	8	88.1	20.5	90.3	0	10
2	90	6	10	224.2	35.8	226.7	5	10
3	85	3	8	165	34.8	168.5	0	5
4	80	14	10	170	35.1	173.7	5	10
5	90	14	6	68.8	12.1	69.9	0	5
6	80	6	6	4.4	1.3	4.5	5	30
7	93.5	10	8	154.7	24.5	156.8	0	0
8	90	14	10	57.8	10.4	58.7	0	5
9	85	10	11.5	218.5	48.9	223.9	10	20
10	85	10	4.5	8.0	2.2	9.4	10	10
11	80	14	6	33.4	7.9	34.3	5	5
12	85	10	8	121.3	26.4	124.1	5	10
13	90	6	6	77.4	13.0	23.9	0	0
14	80	6	10	140.8	32.6	163.2	5	20
15	85	10	8	23.5	23.5	114.0	10	15
16	85	10	8	98.7	22.2	101.0	15	30
17	76.5	10	8	104.4	23.3	106.9	0	10

*Note*: *X*_1_: temperature (°C); *X*_2_: time (min); *X*_3_: rice flour amount (%); *Y*_1_: *G*′ (Pa); *Y*_2_: *G*″ (Pa); *Y*_3_: G*(Pa); *Y*_4_: 1st day phase separation (%); *Y*_5_: 7th day phase separation (%).

**TABLE 2:** Experimental design and data under various conditions of whole‐fat cow's milk rice pudding.

Run	Independent variables			Responses				
	*X*_1_	*X*_2_	*X*_3_	*Y*_1_	*Y*_2_	*Y*_3_	*Y*_4_	*Y*_5_
1	85	17	8	96.7	20.0	98.9	0	0
2	90	6	10	46.7	12.2	48.2	0	0
3	76.5	10	8	18.4	14.1	23.2	5	0
4	85	3.5	8	15.9	6.8	17.3	5	5
5	90	14	6	13.1	10.5	16.8	0	0
6	85	10	8	79.7	18.3	82.2	15	15
7	85	10	8	20.5	6.5	21.5	10	10
8	85	10	4.5	4.7	5.4	6.6	10	15
9	80	14	6	13.1	11.2	17.2	5	5
10	85	10	11.5	116.6	23.0	118.8	10	10
11	90	14	10	30.1	9.2	32.1	0	0
12	80	6	10	97.0	26.2	100.3	5	10
13	80	14	10	75.6	19.4	78.1	5	5
14	80	6	6	19.6	12.4	23.2	5	5
15	93.5	10	8	39.2	9.8	40.4	0	0
16	85	10	8	28.7	8.6	30.1	15	15
17	90	6	6	6.6	7.8	10.2	0	0

*Note*: *X*_1_: temperature (°C); *X*_2_: time (min); *X*_3_: rice flour amount (%); *Y*_1_: *G*′ (Pa); *Y*_2_: *G*″ (Pa); *Y*_3_: G*(Pa); *Y*_4_: 1st day phase separation (%); *Y*_5_: 7th day phase separation (%).

The non‐significant lack‐of‐fit for all investigated variables shows that the multinomial models accurately predicted the corresponding responses. As presented in Table 3, the effect of temperature on the phase separation for both cow's milk and oat milk rice pudding was found to be quadratic, while the influence of rice flour amount had a linear effect on *G′*, *G″*, and G* for cow's milk rice pudding. The *R* ^2^ values for whole‐fat cow's milk rice pudding were 0.70, 0.80, 0.83, 0.90, and 0.85, for *G*′, *G*″, and G* and phase separation on the 1st and 7th days, respectively. In the case of oat milk rice pudding, the *R* ^2^ values were 0.75, 0.90, 0.93, 0.80, and 0.85, for *G*′, *G*″, and G* and phase separation on the 1st and 7th days, respectively.

**TABLE 3:** Predictive model relation between independent and response variables.

	Response	Factors^a^	*p*‐value	Model lack of fit	Model equation
Oat milk rice pudding	*G*′	*X*_3_	.0005	0.72^ns^	Linear 0.0024^b^
	*G*″	*X*_3_	.0001	0.11^ns^	2FI 0.0004^b^
		*X*_1_ × *X*_3_	.043		
	G*	*X*_2_	.014	0.17^ns^	2FI 0.0001^b^
		*X*_3_	.0001		
		*X*_1_ × *X*_2_	.011		
		*X*_1_ × *X*_3_	.045		
		*X*_1_ × *X*_3_	.023		
	1st day phase separation	*X*_1_	.012	0.64^ns^	Quadratic 0.0007^b^
		*X*_1_^2^	.0003		
		*X*_2_^2^	.006		
	7th day phase separation	*X*_1_	.004	0.83^ns^	Quadratic 0.03^b^
		*X*_1_ × *X*_2_	.03		
		*X*_1_^2^	.01		
Whole‐fat cow's milk rice pudding	*G*′	*X*_3_	.002	0.76^ns^	Linear 0.009^b^
	*G*″	*X*_3_	.007	0.84^ns^	Linear 0.014^b^
	G*	*X*_3_	.0018	0.78^ns^	Linear 0.01^b^
	1st day phase separation	$X_{1}^{2}$	.0006	0.59^ns^	Quadratic 0.016^b^
		$X_{2}^{2}$	.0017		
	7th day phase separation	$X_{1}^{2}$	.002	0.43^ns^	Quadratic 0.032^b^
		$X_{2}^{2}$	.005		

^a^ *X*_1_: temperature (°C); *X*_2_: time (min); *X*_3_: rice flour amount (%).

^b^ Significant at 0.05 level.

Before optimization, models were discussed to select the factors with significant effects. Subsequently, these chosen factors were evaluated in the optimization section, as outlined by Karimidastjerd (2020). A comparison between predicted and actual values for the response variables indicated that the polynomial regression models were suitable for determining the optimum conditions to produce cow's milk and plant‐based milk rice puddings with similar rheological properties to commercial rice pudding (*G′*: 62.2 Pa, *G″*: 24.2 Pa, and G*: 54.8 Pa) and the lowest phase separation. The optimal conditions were determined as 90°C, 12.5 min and 6.5% rice flour for both oat milk and whole‐fat cow's milk rice puddings when the independent factors were within the range. The desirability for the optimal conditions was calculated to be 85% for oat milk rice pudding and 87% for whole‐fat cow's milk rice pudding.

To validate the predicted values and observed values for the model, three replications were conducted under the optimum conditions listed in Table 4. Inconsistency between the predicted and observed values of viscoelastic parameters might arise from significant variations in the measured data related to viscoelastic properties, which the Design Expert program was linked to optimizing (Karimidastjerd & Kilic‐Akyilmaz, 2021).

**TABLE 4:** Predicted and validated values for whole‐fat cow's milk and oat milk rice puddings under optimum conditions^a^.

Pudding type	*G*′ (Pa)	*G*″ (Pa)	G* (Pa)	Phase separation (1st day)	Phase separation (7th day)
Oat milk rice pudding					
Predicted	78	18.9	106	0	0
Validated	45.3 ± 0.01	9.6 ± 0.0	45.9 ± 0.01	0.8 ± 0.0	0.2 ± 0.0
Whole‐fat milk rice pudding					
Predicted	62.2	13.9	63.8	2.0	1.2
Validated	34.7 ± 0.01	8.1 ± 0.01	40.5 ± 0.01	0.0 ± 0.0	0.0 ± 0.0

^a^ Optimum conditions: 90°C, 12.5 min, 6.5% rice flour.

### 3.2 Proximate composition

The chemical composition of pudding samples is presented in Table 5. There was no significant difference in the dry matter content between dairy, soya, and oat milks, all of which were higher than other plant‐based rice puddings. Protein values ranged from 0.73 ± 0.03% (for coconut milk rice pudding) to 5.05 ± 0.48% (for whole‐fat cow's milk rice pudding). The protein content of soya milk rice pudding did not significantly differ from that of dairy‐based puddings, which had a higher protein content compared to other puddings. Significant differences were observed in terms of oil content, with the highest oil content found in coconut milk rice pudding (3.29 ± 0.16%) and the lowest in low‐fat cow's milk rice pudding (1.89 ± 0.02%), while other pudding sources demonstrated similar oil content. The ash content of pudding samples ranged from 0.13 ± 0.04% for oat milk rice pudding to 0.89 ± 0.16 for whole‐fat cow's milk rice pudding. Rincon et al. (2020) reported that coconut milk had 1.04% protein, 0.32% ash, and 7.42% oil content. They also showed that soya and almond milks contained 2.92% and 0.42% protein, 1.67% and 1.04% oil, respectively. Studies conducted by Jeske et al. (2017) and Singhal et al. (2017) indicated that the protein content of commercial coconut milk was lower than 1%, consistent with our results. The plant‐based milks used in this study were also commercial products. Differences between the literature and our results could be attributed to variations in the extraction process conditions and the use of different raw materials.

**TABLE 5:** Chemical composition and color parameters of rice puddings at optimized conditions prepared by Thermomix®*.

Pudding samples	Energy (kcal)	Oil (%)	Protein (%)	Ash (%)	Carbohydrate (%)	Dry matter (%)	L*	a*	b*	BI
Almond milk rice pudding	67	2.38 ± 0.11^b^	1.15 ± 0.07^b^	0.45 ± 0.01^c^	10.16 ± 0.18	14.13 ± 1.27^c^	75.4 ± 0.01^d^	−1.5 ± 0.01^b^	9.7 ± 0.01^c^	11.96 ± 0.02^c^
Oat milk rice pudding	83	2.13 ± 0.08^b^	0.92 ± 0.07^c^	0.13 ± 0.04^d^	15.08 ± 0.05	18.27 ± 1.18^a^	75.1 ± 0.02^d^	−1.1 ± 0.01^b^	10.8 ± 0.01^c^	13.94 ± 0.03^c^
Pistachio milk rice pudding	74	1.98 ± 0.26^bc^	1.10 ± 0.21^b^	0.47 ± 0.05^b^	12.93 ± 0.52	16.48 ± 2.14^b^	61.8 ± 0.04^e^	2.1 ± 0.06^a^	17.2 ± 0.03^a^	34.40 ± 0.16^a^
Soya milk rice pudding	84	1.91 ± 0.18^b^	4.50 ± 0.11^a^	0.35 ± 0.02^c^	12.21 ± 0.55	18.96 ± 2.56^a^	76.8 ± 0.05^b^	−3.0 ± 0.01^c^	13.9 ± 0.01^b^	16.48 ± 0.00^b^
Coconut milk rice pudding	75	3.29 ± 0.16^a^	0.73 ± 0.03^d^	0.35 ± 0.05^c^	10.53 ± 0.09	14.91 ± 1.24^b^	76.2 ± 0.11^c^	−1.2 ± 0.01^b^	0.1 ± 0.02^d^	−1.01 ± 0.01^e^
Whole‐fat cow's milk rice pudding	98	2.06 ± 0.03^b^	5.05 ± 0.48^a^	0.69 ± 0.05^a^	14.86 ± 0.49	22.66 ± 2.27^a^	85.9 ± 0.02^a^	−3.1 ± 0.01^c^	6.9 ± 0.24^c^	5.51 ± 0.29^d^
Low‐fat cow's milk rice pudding	90	1.89 ± 0.02^c^	4.76 ± 0.07^a^	0.89 ± 0.16^a^	13.52 ± 0.21	21.06 ± 1.01^a^	83.3 ± 0.04^a^	−3.6 ± 0.05^c^	6.1 ± 0.04^c^	4.19 ± 0.01^d^

* Mean ± standard deviation (*n* = 3); means with different superscript letters in the same column are statistically different (*p* < .05).

### 3.3 Instrumental color parameters

Color is another physicochemical property mainly influenced by different formulations, processing conditions, and storage times. The color parameter was measured using the Hunter instrument, with brightness (L*) for pudding samples ranging from 61.8 ± 0.04 for pistachio milk rice pudding to 85.9 ± 0.02 for whole‐fat cow's milk rice pudding (Table 5). The variation in color between samples can be attributed to the differences in the natural color of raw materials and the interaction of pigments with sugar and rice flour. Jeske et al. (2017) reported that the whiteness index of commercial almond, soya, and quinoa milks exceeded 71.3, which aligns with our results. The lowest L* value was observed in pistachio milk rice pudding, due to the natural color of the pistachios, which had an L* value of 70.21 ± 0.95 (Ling et al., 2016). Whole‐fat and low‐fat cow's milk rice puddings had higher L* values compared to plant‐based rice puddings due to the natural white color of cow's milk. The yellowness value (b*) for all samples was positive, indicating that the appearance color of all puddings leaned toward yellow. It was observed that most of the pudding samples had a generally negative redness value (a*), except for pistachio milk rice pudding, which had a positive a* value. In terms of yellowness (b*) values, pistachio rice pudding (17.2) exhibited the highest value (*p* < .05), followed by soya rice pudding (13.9). No significant differences were observed (*p* > .05) among the b* values for the other plant‐based rice puddings and milk‐based ones, except for coconut milk, which had the lowest b* value (0.1) (*p* < .05). Notably, there was no difference in color values between both dairy rice puddings, while plant‐based rice puddings showed significant differences in color values due to their natural colors (*p* < .05). These differences are primarily attributed to the variations in color among milk types, as we measured L*, a*, and b * values for all milk types. According to L* values, the milks ranked from lightest to darkest were whole milk (88.6) > low‐fat milk (86.1) > oat milk (79.4) > almond milk (73.8) > coconut milk (71.3) > soya milk (68.6) > pistachio milk (63.3). The a* values were positive for pistachio milk (2.51) and soya milk (0.31) and negative for the other milk types. The b* values were highest for pistachio (17.9), followed by oat milk (13.6), soya milk (7.7), almond milk (88.9), whole milk (6.4), low‐fat milk (3.6), and coconut milk (1.6), which had the lowest b* values.

The BI is another color parameter used to evaluate the physical quality of processed food. According to the BI results (Table 5), pistachio milk pudding had the highest BI value (34.41), followed by soya milk (16.48), oat milk (13.94), and almond milk (11.96) rice puddings in descending order. In contrast, coconut milk rice pudding exhibited the lowest BI value (−1.009) (*p* < .05).

The Maillard reaction could potentially explain the variation in colors in puddings, although under optimized conditions, the process occurs similarly for all samples. Valine, leucine, isoleucine, phenylalanine, and cysteine are the amino acids that participate in the Millard reaction (Pripis‐Nicolau et al., 2000), and these components are present in rice flour (NutrientOptimiser, 2023). Under the optimized conditions, all samples contained 6.5% (w/v) rice flour and the same amount of sugar (5%, w/v). While rice pudding samples with plant‐based milk proteins had lower protein contents than milk‐based rice puddings, the color parameters and BI results of milk‐based samples were not significantly different from those of plant‐based ones (Table 5). The temperature was maintained at 90°C under optimized conditions for all samples, and the Millard reaction occurs in the temperature range of 55–100°C, depending on the water content, high protein/sugar concentration, and pH >7.0 (Pripis‐Nicolau et al., 2000). The pH values of all samples measured in the neutral range were between 6.6 and 7.6. Therefore, we concluded that a very low rate of Millard reaction could occur over one night of storage at +4°C in the samples.

### 3.4 Rheological properties

Rice puddings are considered as semi‐solid foods that develop a firm texture through the interaction between proteins and starch during the heating and cooling process. The rheological properties of plant‐based rice pudding can be significantly different from those of dairy pudding due to the presence of certain hydrocolloids in plant‐based milks (Alamprese & Mariotti, 2011). Rice starch, as the primary carbohydrate in pudding, plays a crucial role in influencing the rheological properties, depending on botanical type, concentration, microstructure, and gelatinization conditions (Alamprese & Mariotti, 2011; Moin et al., 2017; Vélez‐Ruiz et al., 2006). Another key ingredient in rice pudding is sugar, which reduces water activity and increases the gelatinization temperature (Alamprese & Mariotti, 2011).

**FIGURE 1:**
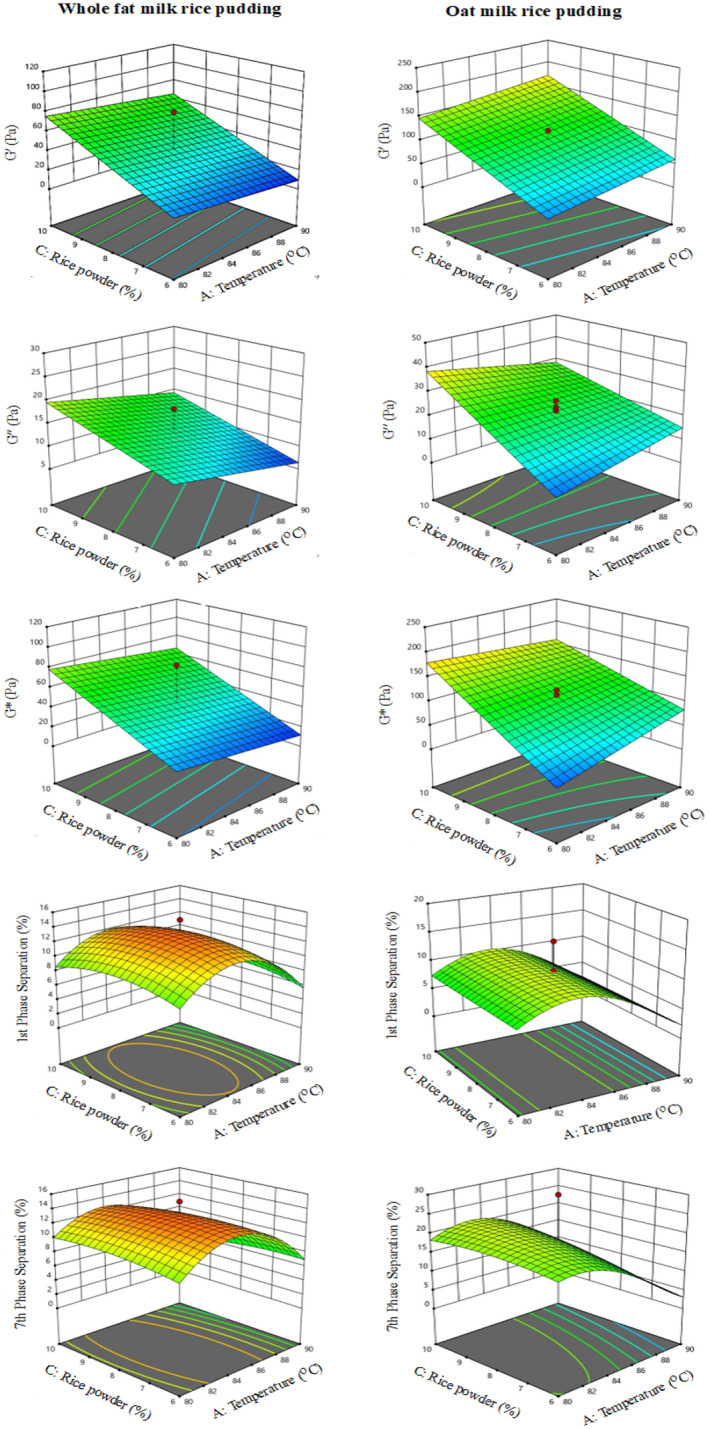
3D response surface plots showing the combined effects of rice flour and temperature on measured factors in whole fat and oat milk rice puddings.

The linear viscoelastic region was observed at 1 Hz within the range of 0.1–100 Hz at 0.5 Pa (Figure 1). As expected, *G′* > *G″* and the loss tangent (*δ = G″*/*G′*) was found to be <1 for all samples, indicating that all pudding samples exhibited behavior consistent with that of a soft gel. For comparing the pudding samples, *G′*, *G″*, G*, and tan *δ* values at 1 Hz frequency for oat and whole fat milk‐based rice puddings are provided in Table 3. The best models for *G′*, *G″*, and G* values of whole‐fat milk rice pudding samples were the linear model, and for the *G′* property of oat milk rice pudding. The best model for *G″* and G* rheological properties of oat milk rice pudding samples was the 2FI model (Table 3). Additionally, under optimized conditions, the rheological properties of the remaining 5 different rice puddings were measured and reported in Table 6.

**TABLE 6:** Rheological and textural parameters of rice puddings at optimized conditions prepared by Thermomix®^†^,^‡^.

Pudding samples	*G*′(Pa)	*G*″(Pa)	G*(Pa)	Tan *δ*	Hardness (*N*)	Gumminess (*N*)	Cohesiveness	Chewiness (Nmm)
Almond milk rice pudding	65.45 ± 1.3^c^	10.43 ± 0.3^d^	64.11 ± 3.3^c^	0.16	0.54 ± 0.015^c^	0.24 ± 0.002^b^	0.40 ± 0.003^b^	2.19 ± 0.05^a^
Oat milk rice pudding	45.3 ± 0.4^d^	9.60 ± 0.1^d^	45.85 ± 0.3^d^	0.22	0.34 ± 0.004^d^	0.17 ± 0.002^c^	0.47 ± 0.01^a^	1.60 ± 0.01^b^
Pistachio milk rice pudding	61.65 ± 2.2^c^	9.53 ± 2.4^d^	57.16 ± 2.9^cd^	0.15	0.33 ± 0.003^d^	0.15 ± 0.00^c^	0.37 ± 0.011^b^	1.28 ± 0.02^c^
Soya milk rice pudding	122.50 ± 2.5^a^	29.55 ± 0.7^a^	129.30 ± 2.4^a^	0.25	0.67 ± 0.003^b^	0.32 ± 0.003^a^	0.38 ± 0.004^b^	2.68 ± 0.05^a^
Coconut milk rice pudding	79.10 ± 1.2^b^	15.6 ± 2.6^c^	69.50 ± 0.6^c^	0.20	0.41 ± 0.007^d^	0.22 ± 0.003^b^	0.44 ± 0.012^a^	1.82 ± 0.02^b^
Whole‐fat cow's milk rice pudding	34.65 ± 4.3^e^	8.10 ± 1.9^d^	40.50 ± 0.4^d^	0.24	0.62 ± 0.005^c^	0.34 ± 0.031^a^	0.45 ± 0.02^a^	2.92 ± 0.28^a^
Low‐fat cow's milk rice pudding	83.70 ± 3.7^b^	23.05 ± 0.4^b^	80.91 ± 0.1^b^	0.28	0.75 ± 0.012^a^	0.36 ± 0.007^a^	0.44 ± 0.01^a^	3.12 ± 0.10^a^

^†^ Mean ± standard deviation (*n* = 3); means with different superscript letters in the same column are statistically different (*p* < .05).

^‡^ Rheological results were obtained at a frequency of 1 Hz under optimized conditions.

A larger *G′* value indicates stronger interactions between particles (Toker et al., 2013), while a lower *G′* value suggests weaker bonds within the matrix, potentially leading to breakage (Liu et al., 2014). Soya milk rice pudding exhibited the highest *G*′, *G*″, and G* values, indicating a more solid‐like behavior. Conversely, the lowest *G′*, *G″*, and G* values were observed in whole‐fat cow's milk rice pudding; however, the tan *δ* value was similar to that of soya milk rice pudding. This can be explained by the higher protein content found in cow's milk, which led to interactions with cow's milk proteins and rice starch (Naseer et al., 2022). Notably, while soya milk rice pudding displayed the highest *G*′, *G*″, and G* values, low‐fat cow's milk rice pudding exhibited the highest tan *δ* value.

pH values for both milks and puddings were measured to clarify the pH effect on gel formation during the pudding preparation process. Based on pH measurements, no significant differences were observed in the pH values among all types of milks and puddings. pH values ranged between 6.6 and 7.6 for both milks and puddings. In contrast to Swanson et al. (2012) report, which claimed that soya milk has an alkaline pH value, our results showed that the pH of soya milk was 7.2.

### 3.5 Texture analysis

Textural parameters of seven pudding samples produced under optimized conditions (90°C, 12.5 min, and 6.5% w/v rice flour) were evaluated after 1 day of storage at 4°C. Texture is a quality parameter that encompasses the structural, mechanical, and surface properties of samples. The data presented in Table 6 revealed that low‐fat cow's milk rice pudding exhibited the highest hardness values. Soya milk rice pudding had a statistically higher hardness value when compared with other plant‐based and whole‐fat cow's milk rice puddings. To obtain a desirable rice pudding, its textural properties should exhibit a semi‐solid, gel‐like structure. Alamprese and Mariotti (2011) reported that low temperatures do not allow for the complete gelatinization of starch, and high temperatures can induce the unfolding, denaturation, and aggregation of rice proteins. Therefore, heating rice above the gelatinization temperature (62–82°C) was necessary to obtain the desired structure for puddings. In our study, the heating range fully enabled the gelatinization of starch and the denaturation of proteins in rice flour (Alamprese & Mariotti, 2011; Moin et al., 2017).

The lowest chewiness was observed in pistachio and oat coconut milk rice puddings. The chewiness property of soya milk rice pudding was found to be similar to that of cow's milk rice puddings. Cohesiveness, which relates to adhesion and represents the extent of gel unity, was higher in cow's milk, coconut soya, and oat milk rice puddings compared to others. Oat and pistachio rice puddings exhibited significantly less gumminess than dairy rice puddings (*p* < .05). Soya milk rice pudding had a similar gumminess value to whole‐fat and low‐fat milk rice puddings.

### 3.6 Phase separation

For semi‐solid food matrixes such as pudding, yogurt, and sour cream, stability can be determined through phase separation, which is defined as syneresis and represents a change in quality (Alamprese & Mariotti, 2011; Moin et al., 2017). The amount of water released during storage is a crucial factor influencing product shelf life and, conversely, can impact consumer acceptability. The highest syneresis is reported in desserts with the lowest dry matter content, a result of a weak gel structure (Yarabbi et al., 2023). In dairy rice puddings, the interaction between rice starch and milk proteins forms a desirable structure. Moreover, raw materials such as milk fat, sugar, rice, and processing factors including mixing speed and duration, heating temperature, and heating and cooling processes can affect the stability of dairy puddings (Alamprese & Mariotti, 2011; McClements et al., 2019; Pracham & Thaiudom, 2016).

Phase separation values ranged from 0% to 10% for pudding samples. According to the RSM results provided in Table 3, for both oat milk and whole‐fat milk rice puddings, the quadratic model is suitable on the 1st day and after 7 days of storage. Pistachio milk rice pudding had the highest phase separation at 5% on the first day, which increased to 10% after 7 days of storage at 4°C. Oat, almond, soya, coconut milk rice puddings, and whole‐fat and low‐fat cow's milk rice puddings did not exhibit any phase separation even after 7 days of storage. During dairy pudding production, milk proteins and rice starch components (amylose and amylopectin) interact to form the structure of the pudding (Alamprese & Mariotti, 2011; Ares et al., 2009; Pracham & Thaiudom, 2016; Thaiudom & Pracham, 2018). The dessert made from 100% sprouted quinoa milk, which had the highest amount of dry matter, demonstrated a greater water‐holding capacity compared to the skimmed milk powder dessert (Yarabbi et al., 2023). Puddings made from plant‐based milk were improved by the addition of stabilizers such as gellan, guar, and xanthan gums, resulting in reduced phase separation due to their high water‐holding capacity.

### 3.7 Sensory analysis

**FIGURE 2:**
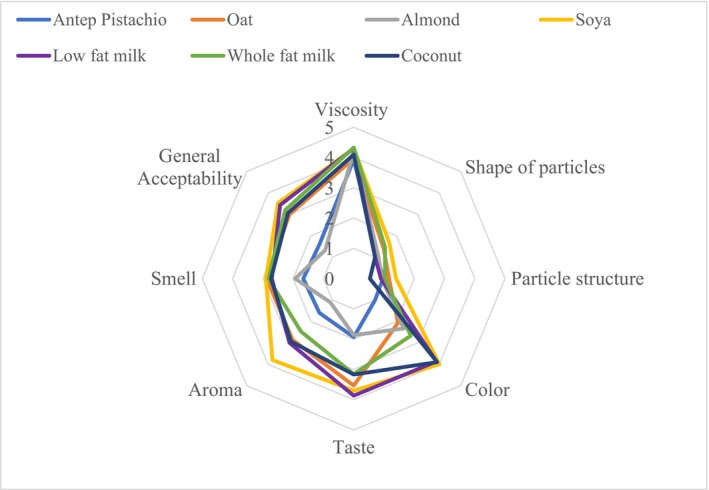
Spider diagram of rice puddings.

To evaluate the sensory characteristics of plant‐based and cow's milk rice puddings, a descriptive method was designed, and results were obtained from 10 trained panelists. The average scores for viscosity, particle structure, shape of particles, color, taste, aroma, smell, and general acceptability are presented in Figure 2. Viscosity, particle size, shape of particles, and color properties were scored using a descriptive scale. The particle structure and shape of particles for all pudding samples were found to be not significantly different (*p* > .05) and were similar to cow's milk rice puddings. Taste, aroma, smell, and general acceptability were evaluated based on a hedonic scale (ranging from extremely dislike to extremely like), and almond and pistachio rice puddings received significantly lower scores than the others (*p* < .05).

Rice pudding made with oat, coconut, and soya milk received the highest overall acceptability among plant‐based milks, although not significantly higher than whole‐fat, low‐fat cow's milk rice puddings. Previous studies related to plant‐based puddings prepared with soya milk (Jung & Joo, 2011), coconut milk (6%), and milk powder received acceptable scores from panelists (Dawane et al., 2010). Sensory evaluation scores for some legume‐based dairy products are similar in terms of color and viscosity but differ in odor and taste (Sethi et al., 2016). Therefore, we can conclude that various rice puddings made from different plant‐based milks may exhibit unique odors, tastes, and even colors despite undergoing the same production process.

Plant‐based rice pudding can be considered a healthy option due to its nutritional properties, such as low allergenic proteins and lactose‐free content. However, researchers have claimed that the occurrence of off‐flavors and other undesirable sensory properties in final products can decrease consumer acceptability (Ares et al., 2009). According to sensory evaluation, oat, coconut, and soya milk rice puddings are liked by consumers as much as dairy puddings. Nevertheless, it is important to evaluate the sensory properties of pudding samples during the storage period.

## 4 CONCLUSION

In the present study, our aim was to develop several plant‐based rice pudding formulations as sustainable, dairy‐free alternatives. We optimized the processing conditions, such as time, temperature, and rice flour amount, using RSM to achieve storage stability and structural quality comparable to dairy rice pudding.

Our findings revealed that optimized processing conditions resulted in minimal phase separation and similar rheological properties compared to dairy‐based rice puddings. All plant‐based rice pudding formulations exhibited negligible phase separation during 7 days of storage, except for pistachio milk rice pudding. Additionally, coconut, oat, and soya milk rice puddings received the highest scores for overall acceptability, demonstrating great potential for industrial production and consumer acceptance of plant‐based rice puddings.

This study contributes valuable insight for the industrial‐scale production of plant‐based rice puddings, which have significant potential for sustainable food production with a minimal number of ingredients, yielding a safe, clean‐labeled, and eco‐friendly dessert product that also caters to the demands of the vegan community. Soya milk, in particular, appears to be a promising alternative for the commercial production of plant‐based rice pudding, given its desirable rheological, textural, and sensory properties under optimized conditions. The outcomes of this study can facilitate the commercialization of new vegan rice puddings, expand product varieties in the market, and promote the use of tools like the Thermomix®, which is a multifunctional tool primarily used in gastronomy. Further studies may explore the shelf life of plant‐based alternatives and enhance sensory acceptability through the incorporation of various hydrocolloids and flavoring agents.

## AUTHOR CONTRIBUTIONS

**Atefeh Karimidastjerd:** Formal analysis (equal); investigation (equal); methodology (equal); writing – original draft (equal). **Zehra Gulsunoglu‐Konuskan:** Formal analysis (equal); investigation (equal); methodology (equal); writing – original draft (equal). **Emine OLUM:** Writing – original draft (supporting). **Omer Said Toker:** Writing – review and editing (supporting).

## CONFLICT OF INTEREST STATEMENT

All authors declare no conflicts of interest.

## Data Availability

Research data are available within the article.

## References

[fsn33872-bib-0001] Alamprese, C. , & Mariotti, M. (2011). Effects of different milk substitutes on pasting, rheological and textural properties of puddings. LWT‐Food Science and Technology, 44(10), 2019–2025.

[fsn33872-bib-0002] Alamri, M. S. , Mohamed, A. A. , & Hussain, S. (2014). High‐fiber date pits pudding: Formulation, processing, and textural properties. European Food Research and Technology, 239(5), 755–763.

[fsn33872-bib-0003] Amend, C. , & Cavagnaro, E. (2021). Can social norms motivate Thermomix® users to eat sustainably? Research in Hospitality Management, 11(2), 121–135.

[fsn33872-bib-0004] AOAC . (1990). Ash of dried milk, method no: 945.46. In K. Helrich (Ed.), Official methods of analysis of the AOAC (15th ed., p. 807). AOAC International.

[fsn33872-bib-0005] AOAC . (1995). Moisture in dried milk, method no: 927.05. In P. Cunniff (Ed.), Official methods of analysis of AOAC international (16th ed., p. 2). AOAC International.

[fsn33872-bib-1005] AOAC . (2000). Official methods of analysis (17th ed., The Association of Official Analytical Chemists.

[fsn33872-bib-0006] AOAC . (2002). Nitrogen (total) in milk Kjeldahl method, method no: 991.20. In W. Horwitz (Ed.), Official methods of analysis of the AOAC international (17th ed., p. 11). AOAC International.

[fsn33872-bib-0007] Ares, G. , Baixauli, R. , Sanz, T. , Varela, P. , & Salvador, A. (2009). New functional fibre in milk puddings: Effect on sensory properties and consumers' acceptability. LWT‐Food Science and Technology, 42(3), 710–716.

[fsn33872-bib-0008] Astolfi, M. L. , Marconi, E. , Protano, C. , & Canepari, S. (2020). Comparative elemental analysis of dairy milk and plant‐based milk alternatives. Food Control, 116, 107327.

[fsn33872-bib-0009] Bähler, B. , Ruf, T. , Samudrala, R. , Schenkel, P. , & Hinrichs, J. (2016). Systematic approach to study temperature and time effects on yield of pasta filata cheese. International Journal of Dairy Technology, 69(2), 184–190.

[fsn33872-bib-0010] Chandan, R. C. , & Kilara, A. (2008). Puddings and dairy‐based desserts. In Dairy processing & quality assurance (pp. 387–410). Wiley.

[fsn33872-bib-0011] Dawane, D. D. , Ranveer, R. C. , & Nagargoje, K. D. (2010). Utilization of tender coconut (*Cocus nucifera* L.) milk in the preparation of pudding. Food Science Research Journal, 1(2), 111–115.

[fsn33872-bib-0012] Freire, M. , Bou, R. , Cofrades, S. , Solas, M. T. , & Jiménez‐Colmenero, F. (2016). Double emulsions to improve frankfurter lipid content: Impact of perilla oil and pork backfat. Journal of the Science of Food and Agriculture, 96(3), 900–908. 25752293 10.1002/jsfa.7163

[fsn33872-bib-0013] Gharibzahedi, S. M. T. (2018). Favorite and traditional rice flour–based puddings, breads, and pastries in the north of Iran: A review. Journal of Ethnic Foods, 5(2), 105–113.

[fsn33872-bib-0014] Ghasemlou, M. , Khodaiyan, F. , Jahanbin, K. , Gharibzahedi, S. M. T. , & Taheri, S. (2012). Structural investigation and response surface optimisation for improvement of kefiran production yield from a low‐cost culture medium. Food Chemistry, 133(2), 383–389. 25683410 10.1016/j.foodchem.2012.01.046

[fsn33872-bib-0015] Goufo, P. , & Trindade, H. (2014). Rice antioxidants: Phenolic acids, flavonoids, anthocyanins, proanthocyanidins, tocopherols, tocotrienols, γ‐oryzanol, and phytic acid. Food Science & Nutrition, 2(2), 75–104. 24804068 10.1002/fsn3.86 PMC3959956

[fsn33872-bib-0016] Hendek Ertop, M. , Atasoya, R. , & Akın, Ş. S. (2019). Evaluation of taro [*Colocasia Esculenta* (L.) Schott] flour as a hydrocolloid on the physicochemical, rheological, and sensorial properties of milk pudding. Journal of Food Processing and Preservation, 43(10), e14103.

[fsn33872-bib-0017] Jeske, S. , Zannini, E. , & Arendt, E. K. (2017). Evaluation of physicochemical and glycaemic properties of commercial plant‐based milk substitutes. Plant Foods for Human Nutrition, 72(1), 26–33. 27817089 10.1007/s11130-016-0583-0 PMC5325842

[fsn33872-bib-0018] Jung, E. K. , & Joo, N. M. (2011). Optimization of soya pudding using response surface methodology. Journal of the Korean Society of Food Culture, 26(6), 717–726.

[fsn33872-bib-0019] Karimidastjerd, A. (2020). Production of a casinomacropeptide concentrate from sweet whey and usage in a milk‐like drink (doctoral dissertation, Institute of Science and Technology). Istanbul Technical University.

[fsn33872-bib-0020] Karimidastjerd, A. , & Gulsunoglu‐Konuskan, Z. (2021). Health benefits of plant‐based milks as alternatives to conventional milk. In H., Erdal (Ed.), Health & Science (pp. 293–310). Efe Academy.

[fsn33872-bib-0021] Karimidastjerd, A. , & Kilic‐Akyilmaz, M. (2021). Formulation of a low‐protein rice drink fortified with caseinomacropeptide concentrate. Food and Bioproducts Processing, 125, 161–169.

[fsn33872-bib-0022] Kolniak‐Ostek, J. , Oszmiański, J. , & Wojdyło, A. (2013). Effect of l‐ascorbic acid addition on quality, polyphenolic compounds and antioxidant capacity of cloudy apple juices. European Food Research and Technology, 236(5), 777–798.

[fsn33872-bib-0023] Lavelle, C. , This, H. , Kelly, A. L. , & Burke, R. (Eds.). (2021). Handbook of molecular gastronomy: Scientific foundations, educational practices, and culinary applications. Crc Press.

[fsn33872-bib-0024] Lawless, H. T. , & Heymann, H. (2010). Sensory evaluation of food: Principles and practices (Vol. 2). Springer.

[fsn33872-bib-0025] Lim, H. S. , & Narsimhan, G. (2006). Pasting and rheological behavior of soya protein‐based pudding. LWT‐Food Science and Technology, 39(4), 344–350.

[fsn33872-bib-0026] Ling, B. , Hou, L. , Li, R. , & Wang, S. (2016). Storage stability of pistachios as influenced by radio frequency treatments for postharvest disinfestations. Innovative Food Science & Emerging Technologies, 33, 357–364.

[fsn33872-bib-0053] Liu, Z. , Juliano, P. , Williams, R. P. , Niere, J. , & Augustin, M. A. (2014). Ultrasound improves the renneting properties of milk. Ultrasonics Sonochemistry, 21(6), 2131–2137. 24751292 10.1016/j.ultsonch.2014.03.034

[fsn33872-bib-0027] Maskan, M. (2001). Kinetics of colour change of kiwifruits during hot air and microwave drying. Journal of Food Engineering, 48(2), 169–175.

[fsn33872-bib-0028] McClements, D. J. (2020). Development of next‐generation nutritionally fortified plant‐based milk substitutes: Structural design principles. Food, 9(4), 421. 10.3390/foods9040421 PMC7231295 32260061

[fsn33872-bib-0029] McClements, D. J. , Newman, E. , & McClements, I. F. (2019). Plant‐based milks: A review of the science underpinning their design, fabrication, and performance. Comprehensive Reviews in Food Science and Food Safety, 18(6), 2047–2067. 33336952 10.1111/1541-4337.12505

[fsn33872-bib-0030] Moin, A. , Ali, T. M. , & Hasnain, A. (2017). Characterization and utilization of hydroxypropylated rice starches for improving textural and storage properties of rice puddings. International Journal of Biological Macromolecules, 105, 843–851. 28732728 10.1016/j.ijbiomac.2017.07.109

[fsn33872-bib-0031] Müller, K. , Jesdinszki, M. , & Schmid, M. (2017). Modification of functional properties of whey protein isolate nanocomposite films and coatings with nanoclays. Journal of Nanomaterials, 2017, 1–10.

[fsn33872-bib-0054] Naseer, B. , Naik, H. R. , Hussain, S. Z. , Qadri, T. , Dar, B. N. , Amin, T. , Reshi, M. , Shafi, F. , & Fatima, T. (2022). Development of low glycemic index instant Phirni (pudding) mix‐its visco‐thermal, morphological and rheological characterization. Scientific reports, 12(1), 10710. 35739179 10.1038/s41598-022-15060-6 PMC9225996

[fsn33872-bib-0032] Ngamlerst, C. , Prangthip, P. , Leelawat, B. , Supawong, S. , & Vatthanakul, S. (2022). A vital role of high‐pressure processing in the gel forming on new healthy egg pudding through texture, microstructure, and molecular impacts. Food, 11(17), 2555. 10.3390/foods11172555 PMC9454986 36076740

[fsn33872-bib-0033] Ng'ong'ola‐Manani, T. A. , Mwangwela, A. M. , Schüller, R. B. , Østlie, H. M. , & Wicklund, T. (2014). Sensory evaluation and consumer acceptance of naturally and lactic acid bacteria‐fermented pastes of soybeans and soybean–maize blends. Food Science & Nutrition, 2(2), 114–131. 24804070 10.1002/fsn3.82 PMC3959958

[fsn33872-bib-0034] Nugroho, K. P. A. , Rahardjo, M. , & Jovitatera, A. (2018). The optimization of pudding formulation using noni (*Morinda citrifolia* L.) seen from antioxidant content and sensory characteristics. Indonesian. Journal of Agricultural Research, 1(3), 280–288.

[fsn33872-bib-0035] Nunes, M. C. , Batista, P. , Raymundo, A. , Alves, M. M. , & Sousa, I. (2003). Vegetable proteins and milk puddings. Colloids and Surfaces B: Biointerfaces, 31(1–4), 21–29.

[fsn33872-bib-0036] NutrientOptimiser . (2023). Rice flour nutritional value and analysis. https://nutrientoptimiser.com/nutritional‐value‐rice‐flour‐white‐unenriched/

[fsn33872-bib-0037] Okur, Ö. D. (2023). Utilization of natural plant sources in a traditional dairy dessert, Muhallebi. Cogent Food & Agriculture, 9(1), 2200601.

[fsn33872-bib-0039] Plamada, D. , Teleky, B. E. , Nemes, S. A. , Mitrea, L. , Szabo, K. , Călinoiu, L. F. , Pascuta, M. S. , Varvara, R. A. , Ciont, C. , Martău, G. A. , Simon, E. , Barta, G. , Dulf, F. V. , Vodnar, D. C. , & Nitescu, M. (2023). Plant‐based dairy alternatives—A future direction to the milky way. Food, 12(9), 1883. 10.3390/foods12091883 PMC10178229 37174421

[fsn33872-bib-0040] Pracham, S. , & Thaiudom, S. (2016). The effect of protein content in jasmine rice flour on textural and rheological properties of jasmine rice pudding. International Food Research Journal, 23(4), 1379–1388.

[fsn33872-bib-0041] Pripis‐Nicolau, L. , De Revel, G. , Bertrand, A. , & Maujean, A. (2000). Formation of flavor components by the reaction of amino acid and carbonyl compounds in mild conditions. Journal of Agricultural and Food Chemistry, 48(9), 3761–3766. 10995267 10.1021/jf991024w

[fsn33872-bib-0042] Rincon, L. , Botelho, R. B. A. , & de Alencar, E. R. (2020). Development of novel plant‐based milk based on chickpea and coconut. LWT‐ Food Science and Technology, 128, 109479.

[fsn33872-bib-0043] Sethi, S. , Tyagi, S. K. , & Anurag, R. K. (2016). Plant‐based milk alternatives an emerging segment of functional beverages: A review. Journal of Food Science and Technology, 53(9), 3408–3423. 27777447 10.1007/s13197-016-2328-3 PMC5069255

[fsn33872-bib-0044] Singhal, S. , Baker, R. D. , & Baker, S. S. (2017). A comparison of the nutritional value of cow's milk and nondairy beverages. Journal of Pediatric Gastroenterology and Nutrition, 64(5), 799–805. 27540708 10.1097/MPG.0000000000001380

[fsn33872-bib-0045] Swanson, R. B. , McKemie, R. J. , Sabrin, M. D. , & Milly, P. J. (2012). Soy milks as a dairy Milk substitute in prepared food products: Effects on quality and acceptability. Family and Consumer Sciences Research Journal, 40(3), 255–266.

[fsn33872-bib-0046] Tarrega, A. , & Costell, E. (2006). Effect of composition on the rheological behaviour and sensory properties of semisolid dairy dessert. Food Hydrocolloids, 20(6), 914–922.

[fsn33872-bib-0047] Thaiudom, S. , & Pracham, S. (2018). The influence of rice protein content and mixed stabilizers on textural and rheological properties of jasmine rice pudding. Food Hydrocolloids, 76, 204–215.

[fsn33872-bib-0048] Toker, O. S. , Dogan, M. , Canıyılmaz, E. , Ersöz, N. B. , & Kaya, Y. (2013). The effects of different gums and their interactions on the rheological properties of a dairy dessert: A mixture design approach. Food and Bioprocess Technology, 6(4), 896–908.

[fsn33872-bib-0049] Velázquez, A. L. , Vidal, L. , Alcaire, F. , Varela, P. , & Ares, G. (2021). Significant sugar‐reduction in dairy products targeted at children is possible without affecting hedonic perception. International Dairy Journal, 114, 104937.

[fsn33872-bib-0050] Vélez‐Ruiz, J. , Hernando, I. , González‐Tomás, L. , Pérez‐Munuera, I. , Quiles, A. , Tárrega, A. , Lluch, M. A. , & Costell, E. (2006). Rheology and microstructure of custard model systems with cross‐linked waxy maize starch. Flavour and Fragrance Journal, 21(1), 30–36.

[fsn33872-bib-0051] Yarabbi, H. , Roshanak, S. , & Milani, E. (2023). Production of the probiotic dessert containing sprouted quinoa milk and evaluation of physicochemical and microbial properties during storage. Food Science & Nutrition, 11(9), 5596–5608. 37701216 10.1002/fsn3.3517 PMC10494662

[fsn33872-bib-0052] Zargaraan, A. , Saghafi, Z. , Hasandokht Firouz, M. , Fadavi, G. , Ghorbani Gorji, S. , & Mohammadifar, M. A. (2015). Effect of rheological properties on sensory acceptance of two‐model dysphagia‐oriented food products. Journal of Texture Studies, 46(3), 219–226.

